# Disparities in Wait Times for Care Among US Veterans by Race and Ethnicity

**DOI:** 10.1001/jamanetworkopen.2022.52061

**Published:** 2023-01-23

**Authors:** Deborah Gurewich, Erin Beilstein-Wedel, Michael Shwartz, Heather Davila, Amy K. Rosen

**Affiliations:** 1 Center for Health Care Organization and Implementation Research, Veterans Affairs (VA) Boston Healthcare System, Boston, Massachusetts; 2 Department of Medicine, Boston University School of Medicine, Boston, Massachusetts; 3 Center for Access & Delivery Research and Evaluation, VA Iowa City Health Care System, Iowa City, Iowa; 4 General Internal Medicine, University of Iowa Carver College of Medicine, Iowa City; 5 Department of Surgery, Boston University School of Medicine, Boston, Massachusetts

## Abstract

**Importance:**

Prior studies indicate that Black and Hispanic vs White veterans wait longer for care. However, these studies do not capture the COVID-19 pandemic, which caused care access disruptions, nor implementation of the US Department of Veterans Affairs (VA) Maintaining Internal Systems and Strengthening Integrated Outside Networks Act (MISSION), which is intended to improve care access by increasing veterans’ options to use community clinicians.

**Objective:**

To determine whether wait times increased differentially for Black and Hispanic compared with White veterans from the pre–COVID-19 to COVID-19 periods given concurrent MISSION implementation.

**Design, Setting, and Participants:**

This cross-sectional study used data from the VA’s Corporate Data Warehouse for fiscal years 2019 to 2021 (October 1, 2018, to September 30, 2021). Participants included Black, Hispanic, and White veterans with a new consultation for outpatient cardiology and/or orthopedic services during the study period. Multivariable mixed-effects models were used to estimate individual-level adjusted wait times and a likelihood ratio test of the significance of wait time disparity change over time.

**Main Outcomes and Measures:**

Overall mean wait times and facility-level adjusted relative mean wait time ratios.

**Results:**

The study included 1 162 148 veterans (mean [SD] age, 63.4 [14.4] years; 80.8% men). Significant wait time disparities were evident for orthopedic services (eg, Black veterans had wait times 2.09 [95% CI, 1.57-2.61] days longer than those for White veterans) in the pre–COVID-19 period, but not for cardiology services. Mean wait times increased from the pre–COVID-19 to COVID-19 periods for both services for all 3 racial and ethnic groups (eg, Hispanic wait times for cardiology services increased 5.09 [95% CI, 3.62-6.55] days). Wait time disparities for Black veterans (4.10 [95% CI, 2.44-5.19] days) and Hispanic veterans (4.40 [95% CI, 2.76-6.05] days) vs White veterans (3.75 [95% CI, 2.30-5.19] days) increased significantly from the pre–COVID-19 to COVID-19 periods (*P* < .001). During the COVID-19 period, significant disparities were evident for orthopedic services (eg, mean wait times for Hispanic vs White veterans were 1.98 [95% CI, 1.32-2.64] days longer) but not for cardiology services. Although there was variation in wait time ratios across the 140 facilities, only 6 facility wait time ratios were significant during the pre–COVID-19 period and 26 during the COVID-19 period.

**Conclusions and Relevance:**

These findings suggest that wait time disparities increased from the pre–COVID-19 to COVID-19 periods, especially for orthopedic services for both Black and Hispanic veterans, despite MISSION’s goal to improve access. Facility-level analyses identified potential sites that could be targeted to reduce disparities.

## Key Points

**Question:**

Did wait times increase differentially for Black and Hispanic veterans compared with White veterans receiving care from the US Veterans Health Administration during the COVID-19 pandemic?

**Findings:**

This cross-sectional study including 1 162 148 US veterans found that wait time disparities increased significantly from the pre–COVID-19 period to the COVID-19 period for Black and Hispanic veterans.

**Meaning:**

These results suggest that Black and Hispanic veterans experienced greater access barriers to care compared with their White counterparts during the COVID-19 pandemic.

## Teaser

This cross-sectional study including US veterans compares wait times for Black and Hispanic veterans with those for White veterans for outpatient orthopedic and cardiology services from fiscal years 2019 to 2021.

## Introduction

The Institute of Medicine defines access to care as having the timely use of personal health services to achieve the best outcomes.^1^ Delayed and disrupted care can adversely affect morbidity and mortality,^2,3,4,5^ as well as health care utilization and patient experience.^6,7,8,9^ For these reasons, wait time (the amount of time it takes a patient to get an appointment and see a clinician) has emerged as a key indicator of overall health system performance.^10^ It is also why disparities in care access, evident for certain subgroups (eg, Black and Hispanic adults) are concerning.^11,12,13,14^ The disparate impact of the COVID-19 pandemic further brought care access disparities into sharper focus. In a national sample of adults in the US, an estimated 40.9% reported delayed care or avoided care because of the COVID-19 pandemic, with delayed care significantly higher for Black and Hispanic adults.^15^

The US Veterans Health Administration (VHA), which provides health care services to 9 million veterans via VHA medical facilities (hereinafter referred to as facilities) across the country, is not immune to care access challenges. The 2014 wait time scandal revealed excessive delays in care for veterans at some VHA facilities and highlighted the need for the VHA to improve timely access to care, especially for outpatient specialty care services.^16,17^ In response, the VHA adopted several policies aimed at improving care access, most significantly establishing the Veterans Choice Program (VCP) in 2014. The VCP expanded opportunities for eligible veterans to receive care from community clinicians paid for by the VHA (hereinafter referred to as community care). Since implementation of VCP, wait times have declined,^18,19,20^ and by 2017, the VHA generally had shorter wait times than those found in the private sector.^19^

Our own work examining wait times for 5 VHA outpatient specialty care services in 2015 and 2018 also reported declining wait times.^20^ However, we additionally found wait time disparities: Black and Hispanic veterans had longer mean wait times for all 5 outpatient specialty care services studied. Other studies conducted both within and outside the VHA^11,12,13,14,21,22^ have noted similar care access disparities by race and ethnicity. However, these studies, like our own, do not capture the more recent operating environment, specifically the disruption in care access due to the COVID-19 pandemic and the implementation of the 2018 US Department of Veterans Affairs (VA) Maintaining Internal Systems and Strengthening Integrated Outside Networks Act (MISSION), which further expanded veterans’ options to use community care. Although several studies have examined how COVID-19 altered health care patterns and utilization among VA-enrolled veterans,^23,24,25,26,27^ to our knowledge, no study has examined the timeliness of care during COVID-19 or the potential influence of COVID-19 on care access disparities. Thus, the degree to which COVID-19 reduced or exacerbated existing wait time disparities in the post-MISSION era needs further study.

In this study, we evaluated changes in wait times for Black and Hispanic veterans compared with White veterans for outpatient orthopedic and cardiology services from fiscal years 2019 to 2021 (October 1, 2018, through September 30, 2021). We assessed whether there were changes in overall and facility-level wait time disparities during the COVID-19 period) compared with the pre–COVID-19 period. We expected that despite potential care access improvements introduced under MISSION, both wait times and wait time disparities would increase overall during the study period and that the number of facilities with statistically significant disparities would likewise increase.

## Methods

This cross-sectional study was deemed as non–human participant research by the VA Boston Healthcare Research Administration, which waived the need for approval and informed consent. We followed the Strengthening the Reporting of Observational Studies in Epidemiology (STROBE) reporting guideline.

### Data Sources and Sample

We used new patient consultation data (ie, approved requests for clinical services) obtained from the consultation schema in the VA’s Corporate Data Warehouse. We focused on cardiology and orthopedic services because they are 2 of the most frequently used outpatient specialty services in the VHA and community care. To identify new-patient consultations, we used VHA stop codes (ie, identifiers that indicate the type of clinical encounter) and additional free-text searching when needed to clarify type of service. We included data for services delivered in the VHA and those purchased through community care.

We obtained veterans’ sociodemographic information (age, sex, and race and ethnicity), and Nosos risk score (from the VA Health Economics Resource Center) for the fiscal year in which a consultation occurred. The Nosos risk score was developed to characterize the disease burden of the veteran population for the purpose of predicting costs.^28,29^ We extracted veterans’ county code and rurality status from the Planning Systems Support Group’s file, combining rural and highly rural and removing those living in an area categorized as an island. County-level physician workforce by specialty supply was obtained from the Area Health Resource File based on the veterans’ county code in the Planning Systems Support Group’s file. Information on social risks (homelessness and food insecurity) was obtained from the VA’s Health Factor table. We used the 3- to 5-digit institution code associated with each consultation to associate it with a specific VHA facility.

Our study sample consisted of veterans who received a new-patient consultation for 1 or both outpatient specialty services during the study period. We followed recommended methods for measuring VHA wait times, which defines a new-patient consultation as a consultation for a veteran who has not had an encounter within the same stop code in the prior 24 months at the same VA Medical Center.^18^ An individual veteran could be included in the analysis up to 2 times per fiscal year if they had 1 consultation for cardiology and 1 for orthopedics.

### Measures

The primary outcome measure was consultation wait time, defined as the number of days from when a veteran’s clinician requested a consultation for the service to when the appointment for that consultation occurred, which is the recommended method for measuring VHA wait times.^18^ Because means and regression coefficients are sensitive to large outliers, we windsorized consultation wait times, specifically, wait times in the top 1% of the distribution for all 3 fiscal years combined across the 2 stop codes (ie, consultations with wait times >179 days [n = 13 301]).

Two independent variables were of primary interest: (1) veterans’ race and ethnicity and (2) time period (pre–COVID-19 period vs COVID-19 period). We classified veterans as Hispanic, non-Hispanic Black (hereinafter Black), and non-Hispanic White (hereinafter White) based on records. Since the World Health Organization declared COVID-19 a pandemic on March 11, 2020,^30^ we defined the pre–COVID-19 period as October 1, 2018, to March 10, 2020, and the COVID-19 period as March 11, 2020, to September 30, 2021. Although the VHA began implementing MISSION in June 2019, full-scale nationwide implementation occurred in 2020,^31^ closer to when the pandemic began.

Additional covariates associated with wait times were also included in the models: age (mean-centered), sex (men and women), marital status (married, divorced or separated, widowed, single, and unknown), social risk (defined as having housing instability and/or food insecurity), rurality status (rural and urban), concurrent Nosos risk score (mean-centered around a score of 1, where a value of 1 indicates a veteran is expected to have costs that represent the national mean for VHA patients), and priority group.^20,32,33,34,35^ Priority group indicates a veteran’s priority level for enrollment in the VHA. It is based on specific eligibility criteria, including severity of service-connected disabilities and income. Veterans in the lowest priority groups have the highest enrollment priority and are exempt from copayments.^28^ Priority levels were grouped into 3 categories (1-4, 5-6, and 7-8). A final variable in the model was physician supply (defined as the number of cardiologists and orthopedists in the county where the veteran lived), which is also associated with wait times.^36,37^

### Statistical Analysis

Although the unit of analysis was a veteran new-patient consultation, we also report descriptive statistics on the characteristics of veterans in our sample and compare the characteristics of White veterans with those of Black and Hispanic veterans. We report means and counts along with the standardized mean differences (ie, effect sizes) to compare subgroups. We interpreted effect sizes as small (0.20), medium (0.50), and large (0.80).^38^ Because of large sample sizes, all *P* values are statistically significant (*P* < .001).

We ran mixed-effects linear regression models to calculate the adjusted wait times separately for each of the 2 services and 2 minority groups (4 models total). The dependent variable was the number of days (ie, wait time) to consultation. Each model included a random slope for the combined race or ethnicity variable interacted with COVID-19 and a random intercept for facility. We considered 2-sided *P* < .05 to be statistically significant. To determine whether disparities changed between periods, we reran the above models with a single combined variable for race or ethnicity and COVID-19 instead of an interaction term. We then used a likelihood ratio test to check whether the more complex model (with the interaction term) fit better than the simple model without the interaction term. If the likelihood ratio test was not significant (*P* > .05), then we would conclude there was no change in disparities between the 2 periods.

To compare adjusted mean wait times for White veterans with those of Black and Hispanic veterans, we followed the approach described by Kleinman and Norton,^39^ except we calculated adjusted relative mean ratios (not relative risk ratios). For each service and starting with the Black vs White veteran model, we calculated the estimated wait time for each veteran separately for the pre–COVID-19 and COVID-19 periods, first assuming the veteran was White and then assuming the veteran was Black (and then similarly for the Hispanic vs White veteran model). The adjusted mean ratio for the Black vs White or Hispanic vs White veteran analysis was the ratio of the mean wait time under the assumption all veterans were Black or Hispanic divided by the mean wait time under the assumption all veterans were White. (Note, because the entire sample for each stop code was included in both the numerator and denominator of the adjusted mean ratio, the case mix was the same except for the race and ethnicity categories.) To determine whether the relative adjusted mean ratios were statistically significant (different from 1, indicating no disparity), we calculated 1000 cluster bootstrapped samples for each stop code by race or ethnicity combinations, using an extension of the approach used by Centers for Medicare & Medicaid Services when profiling facility performance.^40^ If the set of bootstrapped adjusted mean ratios did not contain 1, then we considered it statistically significant at the 95% level. We used R Studio, version 4.1.2 (R Project for Statistical Computing) to perform the analysis.

Because of small sample sizes at some facilities and the problem of multiple comparisons, the power to detect facility relative mean ratios as statistically significant was low. For these reasons, we interpreted statistical significance not as a formal test of a hypothesis but as a signal suggesting that more in-depth monitoring or evaluation of a specific facility would be useful.

## Results

### Descriptive Results: Study Population Characteristics and Wait Times

A total of 1 162 148 veterans with 1 306 383 distinct consultations (80.8% for men and 19.2% for women; mean [SD] age, 63.4 [14.4] years) were included in the analysis. Black and Hispanic veterans were younger (mean [SD] age, 60.1 [12.9] and 58.2 [16.3] years, respectively) compared with White veterans (mean [SD] age, 64.7 [14.3] years; effect sizes, 0.33 and 0.42, respectively) and more likely to reside in counties with higher physician supply. For example, White veterans resided in counties with a mean (SD) of 39.1 (79.3) cardiologists compared with 81.9 (122.2) (effect size, 0.38) for Black veterans and 80.1 (129.2) (effect size, 0.38) for Hispanic veterans. A higher percentage of White veterans resided in rural areas (44.2%) compared with Black veterans (17.5%; effect size, 0.60) and Hispanic veterans (17.2%; effect size, 0.61).

Our sample included a total of 1 306 383 new patients across 140 facilities (Table 1). Of the consultations, 643 124 were for cardiology (among 630 603 veterans) and 663 259 were for orthopedics (among 649 992 veterans). Unadjusted mean wait time differences across groups (Black, Hispanic, and White veterans) were negligible during the pre–COVID-19 and COVID-19 periods for the 2 services; the largest effect size was less than 0.20.

**Table 1.** Patient Characteristics By Race and Ethnicity

Characteristic	Patient group^a^				Effect size	
	Overall	Black	Hispanic	White	Black vs White	Hispanic vs White
No. of consultations	1 306 383	240 385	91 678	974 320	NA	NA
No. of distinct veterans	1 162 148	216 675	82 613	862 860	NA	NA
Outcome^b^						
Wait time, mean (SD), d						
Overall	34 (31.7)	35.4 (32.1)	34.8 (31.7)	33.6 (31.5)	0.06	0.04
Cardiology						
Pre–COVID-19 period	31.6 (29.0)	31.1 (27.7)	33.9 (28.8)	31.5 (29.3)	0.02	0.08
COVID-19 period	36.7 (36.6)	36.8 (35.8)	39.4 (38.9)	36.5 (36.6)	0.01	0.08
Orthopedics						
Pre–COVID-19 period	31.9 (25.4)	35.5 (27.8)	31.0 (24.5)	31,0 (24.7)	0.17	0.002
COVID-19 period	36.2 (35.0)	38.3 (36.7)	36.3 (34.4)	35.7 (34.5)	0.07	0.02
Covariates						
Age, mean (SD), y	63.4 (14.4)	60.1 (12.9)	58.2 (16.3)	64.7 (14.3)	0.33	0.42
Nosos risk score, concurrent, mean (SD)^c^	1.6 (2.2)	1.9 (2.4)	1.5 (2.1)	1.6 (2.1)	0.14	0.02
No. of specialists providing patient care in 2018, mean (SD)						
Cardiologists	50.0 (94.8)	81.9 (122.2)	80.1 (129.2)	39.1 (79.3)	0.42	0.38
Orthopedists	61.4 (113.7)	97.4 (142.8)	98.7 (157.5)	48.8 (96.3)	0.408	0.38
Sex, No. (%) of consultations						
Men	1 055 306 (80.8)	183 105 (76.2)	74 749 (81.5)	797 452 (81.8)	0.25	0.07
Women	106 842 (9.2)	57 280 (23.8)	16 929 (18.5)	176 868 (18.2)	0.25	0.07
COVID-19	551 695 (47.5)	100 785 (46.5)	38 552 (46.7)	412 358 (47.8)	0.03	0.02
Priority group^d^						
1-4	770 376 (66.3)	156 222 (72.1)	61 038 (73.9)	553 116 (64.1)	0.17	0.21
5-6	250 964 (21.6)	40 175 (18.5)	14 878 (18.0)	195 911 (22.7)	0.10	0.12
7-8	140 808 (12.1)	20 278 (9.4)	6697 (8.1)	113 833 (13.2)	0.12	0.17
Location						
Urban	726 344 (62.5)	178 058 (82.2)	68 198 (82.6)	480 088 (55.6)	0.60	0.61
Rural	433 227 (37.3)	38 005 (17.5)	14 203 (17.2)	381 019 (44.2)	0.60	0.61
Unknown	2577 (0.2)	612 (0.3)	212 (0.3)	1753 (0.2)	0.02	0.01
Marital status						
Married	628 159 (54.1)	95 037 (43.9)	46 178 (55.9)	486 944 (56.4)	0.25	0.01
Divorced	284 777 (24.5)	57 757 (26.7)	18 145 (22.0)	208 875 (24.2)	0.06	0.05
Widowed	50 593 (4.4)	7402 (3.4)	2379 (2.9)	40 812 (4.7)	0.07	0.10
Single	175 888 (15.1)	52 173 (24.1)	14 161 (17.1)	109 554 (12.7)	0.30	0.13
Unknown	22 731 (2.0)	4306 (2.0)	1750 (2.1)	16 675 (1.9)	0.004	0.01
Homeless or risk for homelessness						
No	975 442 (83.9)	174 563 (80.6)	69 366 (84.0)	731 513 (84.8)	0.11	0.02
Yes	49 907 (4.3)	18 952 (8.7)	3635 (4.4)	27 320 (3.2)	0.24	0.07
Unknown	136 799 (11.8)	23 160 (10.7)	9612 (11.6)	104 027 (12.1)	0.04	0.01
Food insecurity						
No	955 428 (82.2)	179 075 (82.6)	67 728 (82.0)	708 625 (82.1)	0.01	0.004
Yes	8025 (0.7)	2768 (1.3)	758 (0.9)	4499 (0.5)	0.08	0.05
Unknown	198 695 (17.1)	34 832 (16.1)	14 127 (17.1)	149 736 (17.4)	0.03	0.01

Abbreviation: NA, not applicable.

^a^ Unless otherwise indicated, data are expressed as No. (%) of veterans. Percentages have been rounded and may not total 100.

^b^ The pre–COVID-19 period was designated as October 1, 2018, to March 10, 2020; the COVID-19 period was designated as March 11, 2020, to September 30, 2021.

^c^ Scores range from 0.2 to 54.9, with higher scores indicating higher disease burden.

^d^ Indicates a veteran’s severity of service-connected disabilities and income level. Veteran priority groups 1 and 2 have the highest enrollment priority and are exempt from copayments. Those in priority groups 3 to 8 have incrementally lower priority and higher copayments.^28^

### Adjusted Mean Wait Times

Adjusted mean wait times during the pre–COVID-19 period for cardiology services did not differ significantly between White vs Black or Hispanic veterans (Table 2 and eTable 1 in Supplement 1). In contrast, compared with orthopedic services for White veterans, both Black and Hispanic veterans had significantly longer adjusted mean wait times (2.09 [95% CI, 1.57-2.61] and 1.30 [95% CI, 0.78-1.81] days, respectively; *P* < .001 for both).

**Table 2.** Adjusted Mean Wait Time by Race and Ethnicity and Time Period^a^

Time period by race and ethnicity^b^	Cardiology		Orthopedics	
	Coefficient (95% CI)	*P* value	Coefficient (95% CI)	*P* value
**Black vs White veterans**				
White veterans, pre–COVID-19 period	1 [Reference]	NA	1 [Reference]	NA
Black veterans, pre–COVID-19 period	0.26 (−0.23 to 0.75)	.30	2.09 (1.57 to 2.61)	<.001
White veterans, COVID-19 period	4.48 (3.40 to 5.56)	<.001	3.75 (2.30 to 5.19)	<.001
Black veterans, pre–COVID-19 period	1 [Reference]	NA	1 [Reference]	NA
Black veterans, COVID-19 period	4.74 (3.40 to 6.08)	<.001	4.10 (2.44 to 5.76)	<.001
White veterans, COVID-19 period	1 [Reference]	NA	1 [Reference]	NA
Black veterans, COVID-19 period	0.53 (−0.12 to 1.18)	.11	2.45 (1.79 to 3.10)	<.001
**Hispanic vs White veterans**				
White veterans, pre–COVID-19 period	1 [Reference]	NA	1 [Reference]	NA
Hispanic veterans, pre–COVID-19 period	0.08 (−0.47 to 0.63)	.78	1.30 (0.78 to 1.81)	<.001
White veterans, COVID-19 period	4.49 (3.40 to 5.57)	<.001	3.71 (2.28 to 5.15)	<.001
Hispanic veterans, pre–COVID-19 period	1 [Reference]	NA	1 [Reference]	NA
Hispanic veterans, COVID-19 period	5.09 (3.62 to 6.55)	<.001	4.40 (2.76 to 6.05)	<.001
White veterans, COVID-19 period	1 [Reference]	NA	1 [Reference]	NA
Hispanic veterans, COVID-19 period	0.68 (−0.10 to 1.45)	.09	1.98 (1.32 to 2.64)	<.001

Abbreviation: NA, not applicable.

^a^ The pre–COVID-19 period was designated as October 1, 2018, to March 10, 2020; the COVID-19 period was designated as March 11, 2020, to September 30, 2021.

^b^ Coefficients from 4 different models per specialty are reported as follows: White veterans in the pre–COVID-19 period (reference group) vs Black and Hispanic veterans in the pre–COVID-19 period; White veterans in the pre–COVID-19 period (reference group) vs White veterans during COVID-19; Black and Hispanic veterans in the pre–COVID-19 period (reference group) vs Black and Hispanic veterans during COVID-19; White veterans during COVID-19 (reference group) vs Black and Hispanic veterans during COVID-19.

Compared with the pre–COVID-19 period, adjusted mean wait times during the COVID-19 period for cardiology services increased by 4.48 (95% CI, 3.40-5.56) days for White veterans, 4.74 (95% CI, 3.40-6.08) days for Black veterans, and 5.09 (95% CI, 3.62-6.55) days for Hispanic veterans (*P* < .001 for all). Adjusted mean wait time for orthopedic services followed a similar pattern, increasing 3.75 (95% CI, 2.30-5.19) days for White veterans, 4.10 (95% CI, 2.44-5.19) days for Black veterans, and 4.40 (95% CI, 2.76-6.05) days for Hispanic veterans (*P* < .001 for all). Adjusted mean wait time disparities for Black and Hispanic veterans compared with those for White veterans also increased significantly for both services. For cardiology services, the wait time disparity grew by 0.27 days (calculated as 0.528 less 0.261) for Black veterans and 0.60 days (calculated as 0.680 less 0.079) for Hispanic veterans vs White veterans; for orthopedic services, the disparity increase was 0.36 days (calculated as 2.450 less 2.093) for Black veterans and 0.69 days (calculated as 1.982 less 1.295) for Hispanic veterans (Table 2). The likelihood ratio test for each set of models was statistically significant (*P* < .001), indicating that the disparity change was significant from the pre–COVID-19 to COVID-19 periods (eTable 2 in Supplement 1).

Despite these increases, as in the pre–COVID-19 period, mean wait times for Black and Hispanic veterans vs White veterans differed significantly only for orthopedic services. Black and Hispanic veterans’ mean wait times exceeded those of White veterans by 2.45 (95% CI, 1.79-3.10) days for Black veterans and 1.98 (95% CI, 1.32-2.64) days for Hispanic veterans during the COVID-19 period (*P* < .001 for both).

### Adjusted Relative Mean Wait Time Ratios

**Figure 1.**
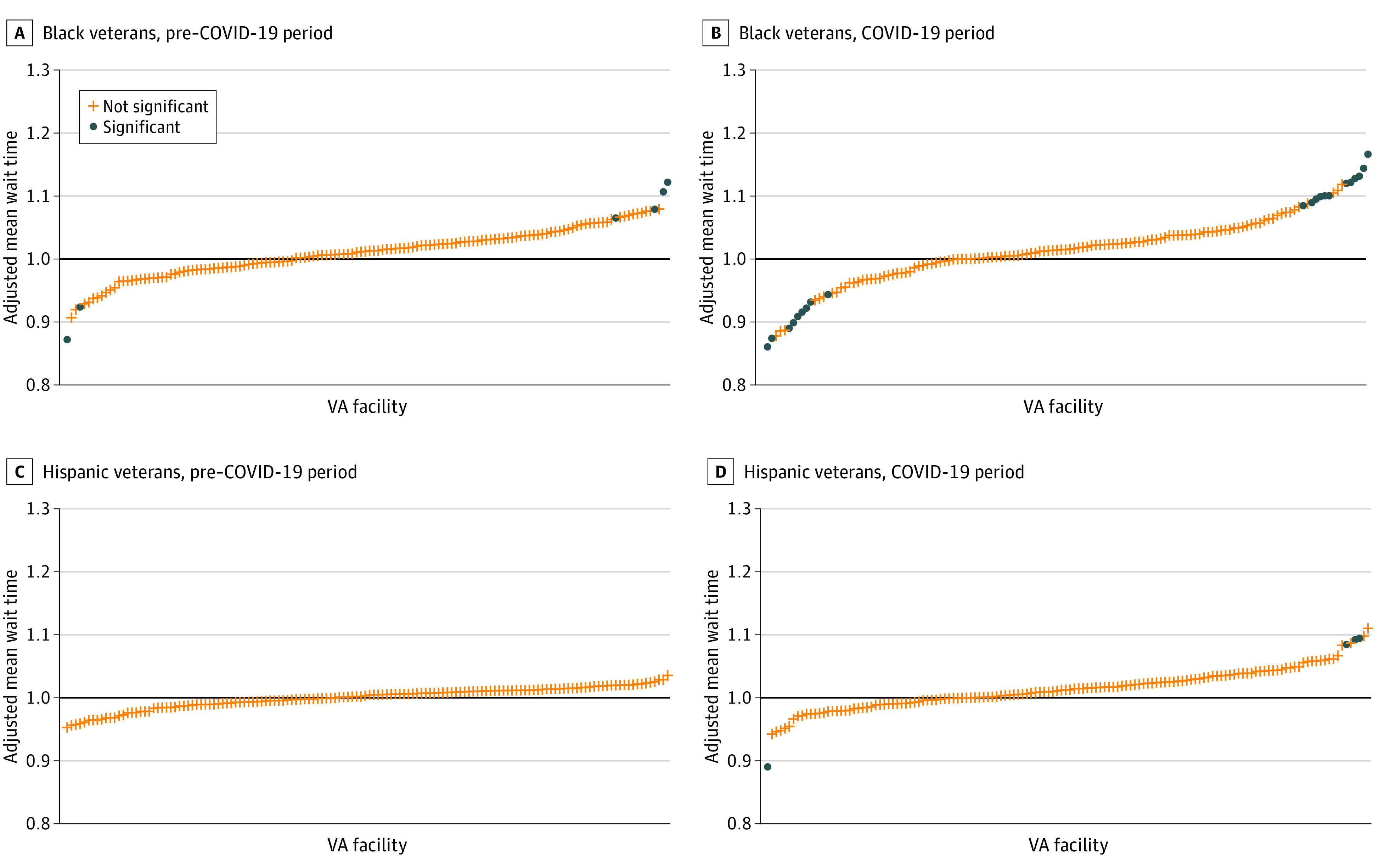
Adjusted Facility Risk Ratios by Race and Ethnicity and Time Period for Cardiology Consultations The pre–COVID-19 period was designated as October 1, 2018, to March 10, 2020; the COVID-19 period was designated as March 11, 2020, to September 30, 2021. VA indicates US Department of Veterans Affairs.

For both specialty services during the pre–COVID-19 period, Figure 1 shows the subset of facilities where the adjusted mean wait time ratios (hereinafter referred to as mean ratio) for Black and Hispanic veterans vs White veterans differed significantly from 1. For cardiology services, the Black-White veteran mean ratio differed significantly at 6 facilities (Black veterans waited longer at 4 facilities; White veterans waited longer at 2). The Hispanic-White veteran mean ratio for cardiology did not differ significantly at any facility. For orthopedic services, neither the Black-White nor Hispanic-White veteran mean ratios differed significantly at any facility during the pre–COVID-19 period.

**Figure 2.**
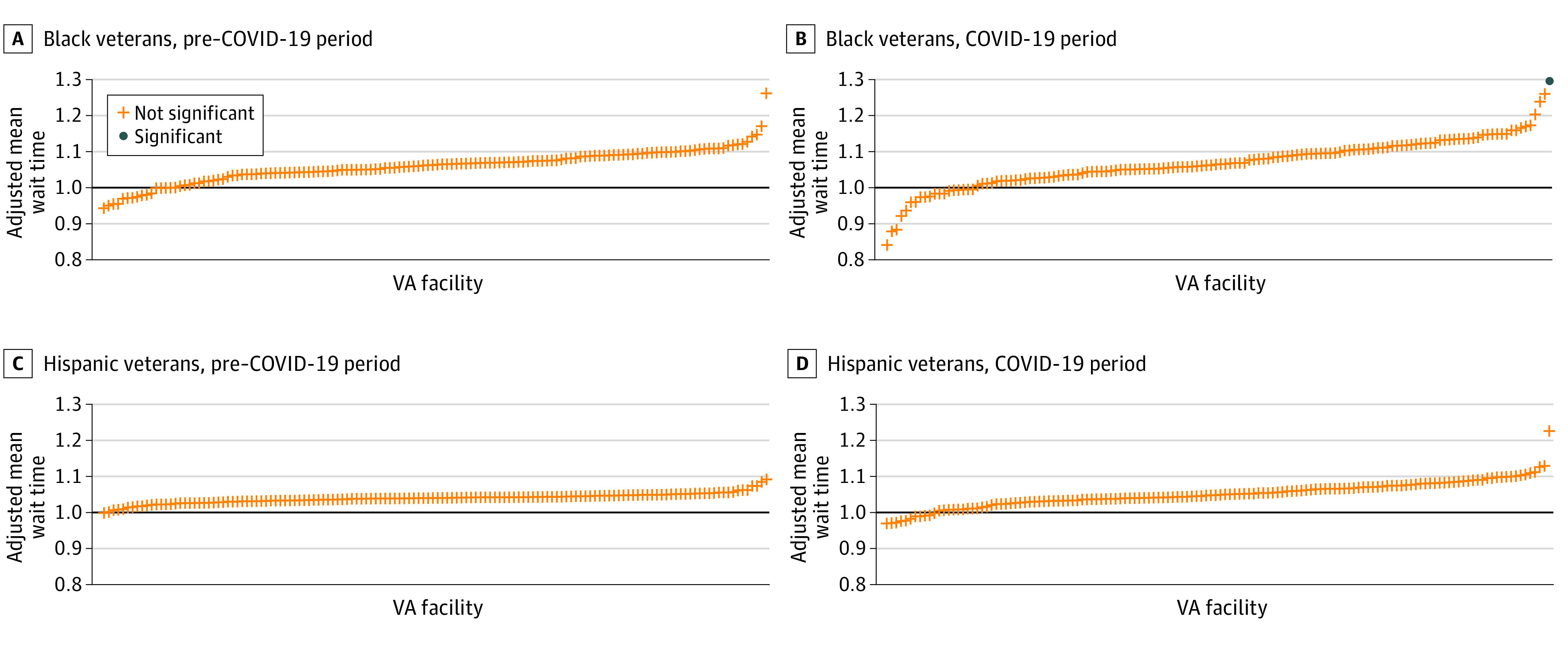
Adjusted Facility Risk Ratios by Race and Ethnicity and Time Period for Orthopedics Consultations The pre–COVID-19 period was designated as October 1, 2018, to March 10, 2020; the COVID-19 period was designated as March 11, 2020, to September 30, 2021. VA indicates US Department of Veterans Affairs.

The number of facilities with mean ratios that differed significantly from 1 increased from the pre–COVID-19 to COVID-19 periods, particularly for cardiology services. The Black-White veteran mean ratio for cardiology services differed significantly at 21 facilities (Figure 2). Of these, Black veterans’ mean wait times were longer at 14 facilities and shorter at 7 facilities compared with wait times for White veterans. The Hispanic-White veteran mean ratio for cardiology services differed significantly at 4 facilities (Hispanic veterans’ mean wait times were longer at 3 facilities and shorter at 1 facility compared with wait times for White veterans). For orthopedic services, the Black-White veteran mean ratio differed significantly at 1 facility, with Black veterans’ mean wait times exceeding those of White veterans. As in the pre–COVID-19 period, the Hispanic-White veteran mean ratio for orthopedic services did not differ significantly at any facility during the COVID-19 period.

## Discussion

There are several important findings from this cross-sectional study. First, as we expected, overall wait times increased substantially during the COVID-19 period. This finding is consistent with other studies^15,23,41,42,43^ and is not surprising, given that substantial numbers of US residents delayed or went without care, particularly early in the pandemic.

Second, we found a significant increase in overall wait time disparities for both Black and Hispanic veterans vs White veterans from the pre–COVID-19 to COVID-19 periods. This finding was consistent with our expectation that despite MISSION, wait time disparities would increase, given the disproportionate impact of COVID-19 on Black and Hispanic communities.^44,45,46,47^ Overall, these increases resulted in notable wait time disparities for Black and Hispanic veterans compared with White veterans for orthopedic services (≤2.5 days longer) but only modest disparities for cardiology (<1-day difference). The relatively modest increase in disparities for cardiology could be due to the rapid expansion in the VHA’s use of telehealth early in the pandemic.^25,26^ Some studies reported higher rates of telehealth use among Black and Hispanic compared with White patients during the COVID-19 pandemic,^48,49,50^ leading to reduced racial and ethnic disparities.^51,52,53^ Regardless, any wait time disparity is concerning, and it will be important for future work to monitor these trends, understand their sources, and implement appropriate interventions as needed.

Third, our national and facility-level findings differed in some respects. For example, nationwide, we did not observe wait time disparities among Black vs White veterans for cardiology during either the pre–COVID-19 or COVID-19 periods. However, we identified 4 facilities during the pre–COVID-19 period and 14 facilities during the COVID-19 period where the mean ratios for cardiology services were significantly different than 1. This finding underscores the critical importance of facility-level analyses for highlighting opportunities to reduce disparities and target quality improvement efforts. It will be important for further work to determine the extent to which these disparities are driven by patient (eg, unequal access to resources needed to use health care services)^54^ or clinician factors (eg, delayed nonvital procedures, discriminatory practices).^55^

The facility-level analyses also identified facilities where the relative mean ratio was significant, but in the opposite direction—that is, where White vs Black or Hispanic veterans waited longer. This finding also suggests a need for further inquiry and interventions. For example, White veterans disproportionately use community care compared with those who identify as Black or other races or ethnicities,^56,57^ and community care is associated with comparatively longer wait times than the VHA.^18,20,58,59^ Thus, facilities where White veterans wait longer than Black or Hispanic veterans may indicate needed improvements in access to community care.

### Limitations

This study has several limitations. First, we examined only wait time disparities for 2 outpatient specialty services; findings for other outpatient services within the VHA may differ. In previous analyses, however, we found that wait times followed similar trends across 5 outpatient specialty services, suggesting there may be consistencies in overall trends.^20^ Second, our measure of wait times was based on VHA administrative data. Although this provided an objective measure of wait times, we were unable to account for veterans’ care perceptions, which could yield different results. Third, although we identified changes in facility-level performance, the measure we used (mean ratios) is one of several measures that could be used to flag sites where interventions may help to reduce wait time disparities.

## Conclusions

In this cross-sectional study, wait time disparities for outpatient cardiology and orthopedic services increased for Black and Hispanic veterans from the pre–COVID-19 to COVID-19 periods compared with White veterans. Specific facilities were identified where wait time disparities were higher, signaling potential opportunities for further investigation and intervention.

## Notes

Supplement 1. **eTable 1.** Full Regression Results **eTable 2.** Log Likelihood Ratio Test Results

Supplement 2.
Data Sharing Statement
