# Supplementary material for: Disparities in Wait Times for Care Among US Veterans by Race and Ethnicity
Source: JAMA Netw Open. 2023 Jan 23;6(1):e2252061. doi: 10.1001/jamanetworkopen.2022.52061 (PMC9871804; doi:10.1001/jamanetworkopen.2022.52061)
Supplement: Supplement 1. — eTable 1. Full Regression Results eTable 2. Log Likelihood Ratio Test Results [file jamanetwopen-e2252061-s001.pdf]

Supplementary Online Content

Gurewich D, Beilstein-Wedel E, Shwartz M, Davila H, Rosen AK. Disparities in wait times for care among US veterans by race and ethnicity. *JAMA Netw Open*. 2023;6(1):e2252061. doi:10.1001/jamanetworkopen.2022.52061

eTable 1. Full Regression Results
eTable 2. Log Likelihood Ratio Test Results

This supplementary material has been provided by the authors to give readers additional information about their work.

© 2023 Gurewich D et al. *JAMA Network Open*.

eTable 1. Full Regression Results

	Cardiology						Orthopedics					
	Blacks			Hispanics			Blacks			Hispanics		
	Coefficient	95% Conf. Int.	p-value	Coefficient	95% Conf. Int.	p-value	Coefficient	95% Conf. Int.	p-value	Coefficient	95% Conf. Int.	p-value
**Race * COVID (ref=White, pre-COVID)**												
Minority, COVID	5.003	(3.70, 6.31)	< 0.001	5.165	(3.83, 6.51)	< 0.001	6.19	(4.62, 7.76)	< 0.001	5.696	(4.10, 7.29)	< 0.001
White, COVID	4.475	(3.40, 5.56)	< 0.001	4.485	(3.40, 5.57)	< 0.001	3.746	(2.30, 5.19)	< 0.001	3.714	(2.28, 5.15)	< 0.001
Minority, pre-COVID	0.261	(-0.23, 0.75)	0.30	0.079	(-0.47, 0.63)	0.78	2.093	(1.57, 2.61)	< 0.001	1.295	(0.78, 1.81)	< 0.001
**Age**	0.127	(0.12, 0.14)	< 0.001	0.132	(0.12, 0.14)	< 0.001	0.03	(0.02, 0.04)	< 0.001	0.023	(0.02, 0.03)	< 0.001
**Female (ref= Male)**	-1.004	(-1.33, -0.68)	< 0.001	-0.916	(-1.30, -0.54)	< 0.001	-0.061	(-0.30, 0.18)	0.62	-0.037	(-0.31, 0.23)	0.79
**Marital Status (ref=Married)**												
Divorced	0.256	(0.05, 0.46)	0.01	0.313	(0.10, 0.53)	0.005	-0.182	(-0.37, 0.00)	0.05	-0.274	(-0.47, -0.08)	0.006
Widowed	-0.022	(-0.40, 0.345)	0.91	0.21	(-0.18, 0.60)	0.30	-0.483	(-0.89, -0.07)	0.02	-0.361	(-0.79, 0.07)	0.10
Single	0.606	(0.34, 0.87)	< 0.001	0.718	(0.42, 1.02)	< 0.001	-0.7	(-0.92, -0.48)	< 0.001	-0.829	(-1.07, -0.59)	< 0.001
Unknown	-0.448	(-1.07, 0.17)	0.16	-0.285	(-0.95, 0.38)	0.40	0.042	(-0.47, 0.56)	0.87	0.073	(-0.47, 0.61)	0.79
**Rurality (ref=Urban)**												

© 2023 Gurewich D et al. *JAMA Network Open*.

Rural	1.417	(1.22, 1.61)	< 0.001	1.399	(1.19, 1.61)	< 0.001	1.125	(0.95, 1.30)	< 0.001	1.097	(0.91, 1.28)	< 0.001
Unknown	8.461	(3.39, 13.53)	0.002	8.555	(2.65, 14.46)	0.005	4.093	(-1.28, 9.47)	0.14	7.036	(0.85, 13.22)	0.03
**Nosos Concurrent**	-0.419	(-0.45, -0.38)	< 0.001	-0.443	(-0.48, -0.40)	< 0.001	-0.493	(-0.53, -0.45)	< 0.001	-0.486	(-0.53, -0.44)	< 0.001
**Priority Group (ref=1-4)**												
5-6	0.096	(-0.11, 0.30)	0.35	0.043	(-0.17, 0.26)	0.70	-0.765	(-0.96, -0.57)	< 0.001	-0.8	(-1.01, -0.59)	< 0.001
7-8	-0.872	(-1.12, -0.62)	< 0.001	-0.92	0 (-1.19, -0.66)	< 0.001	-1.984	(-2.23, -1.74)	< 0.001	-2.035	(-2.29, -1.78)	< 0.001
**Specialty Patient Care, 2018**	-0.01	(-0.01, -0.01)	< 0.001	-0.011	(-0.01, -0.01)	< 0.001	-0.007	(-0.01, -0.01)	< 0.001	-0.008	(-0.01, -0.01)	< 0.001
**Food Insecurity (ref=Not food insecure)**												
Yes, Food insecurity	0.207	(-0.84, 1.25)	0.70	0.743	(-0.48, 1.97)	0.24	0.475	(-0.42, 1.37)	0.30	0.057	(-0.99, 1.10	0.92
Unknown, Food insecurity	-1.461	(-1.89, -1.03)	< 0.001	-1.257	(-1.73, -0.79)	< 0.001	-1.998	(-2.38, -1.61)	< 0.001	-2.035	(-2.45, -1.62)	< 0.001
**Homeless (ref=Not homeless)**												
Homeless or at risk of homelessness	0.292	(-0.14, 0.73)	0.19	0.314	(-0.21, 0.84)	0.25	-0.289	(-0.67, 0.09)	0.13	-0.299	(-0.75, 0.15)	0.19
Unknown	0.685	(0.21, 1.16)	0.005	0.58	(0.06, 1.10)	0.03	0.343	(-0.08, 0.77)	0.12	0.268	(-0.19, 0.72)	0.25
**Constant**	24.118	(22.53, 25.71)	< 0.001	23.811	(22.25, 25.37)	< 0.001	31.218	(29.54, 32.90)	< 0.001	31.724	(30.07, 33.38)	< 0.001

© 2023 Gurewich D et al. *JAMA Network Open*.

eTable 2. Log Likelihood Ratio Test Results

	Cardiology		Orthopedics	
	Blacks	Hispanics	Blacks	Hispanics
Full Model Log Likelihood	-2917610	-2578294	-2912414	-2536090
Simplified Model Log Likelihood	-2918700	-2578674	-2914339	-2536748
Likelihood Ratio Test p-value	<0.001	<0.001	<0.001	<0.001

© 2023 Gurewich D et al. *JAMA Network Open*.
