# Supplementary material for: Disparities in Wait Times for Care Among US Veterans by Race and Ethnicity
Source: JAMA Netw Open. 2023 Jan 23;6(1):e2252061. doi: 10.1001/jamanetworkopen.2022.52061 (PMC9871804; doi:10.1001/jamanetworkopen.2022.52061)
Supplement: Supplement 2. — Data Sharing Statement [file jamanetwopen-e2252061-s002.pdf]

Data Sharing Statement

Gurewich. Disparities in Wait Times for Care Among US Veterans by Race and Ethnicity. *JAMA Netw Open*. Published January 23, 2023. doi:10.1001/jamanetworkopen.2022.52061

Data
Data available: No

Additional Information
Explanation for why data not available: We do not plan to share the data because there are several covariates that risk identifying individual patients, including dates of health care service and homeless status.
